# Endogenous micropeptides as potential diagnostic biomarkers and therapeutic drugs

**DOI:** 10.3389/fphar.2025.1545575

**Published:** 2025-04-08

**Authors:** Aixi Zhong, Shuai Li, Jingxuan Zhang, Jingyuan Zhao, Chenhui Yao

**Affiliations:** ^1^ The First Affiliated Hospital of Dalian Medical University, Dalian, China; ^2^ Dalian Medical University, Dalian, China; ^3^ Zhongshan College of Dalian Medical University, Dalian, China; ^4^ Central Hospital of Dalian University of Technology, Dalian, China

**Keywords:** micropeptides, diagnostic biomarkers, drugs, noncoding RNAs (ncRNAs), peptide

## Abstract

Micropeptides, these small proteins derived from non-coding RNA, typically consist of no more than 100 amino acids in length. Despite the challenges in analysis and identification, their various critical functions within organisms cannot be overlooked. They play a significant role in maintaining energy metabolism balance, regulating the immune system, and influencing the development of tumors, which also gives them a decisive impact on the occurrence and development of various diseases. This review aims to outline the role and potential value of micropeptides, introducing their tissue classification and distribution, biological functions, and mechanisms, with a focus on their potential as diagnostic markers and therapeutic drugs.

## 1 Introduction

Micropeptides are small peptides derive from noncoding RNAs (ncRNAs) and are encoded by small open reading frames (sORFs) with a size of less than 100 amino acids. Micropeptides are short peptide molecules produced internally within the human body, primarily originating from small open reading frames (sORFs) within non-coding RNA (ncRNA) (Vitorino et al., 2021). Despite the human body having approximately 20,000 to 25,000 protein-coding genes, about three-quarters of which can be transcribed, only about 0.02% of these genes have the potential to encode proteins (Djebali et al., 2012). Research indicates that the transcripts outside of protein-coding genes contain a large amount of non-coding RNA (ncRNA). Within these ncRNAs, there may be one or more short open reading frames that could potentially encode a very small protein of less than 100 amino acids, known as a micro-peptide (Chen et al., 2021).

**FIGURE 1:**
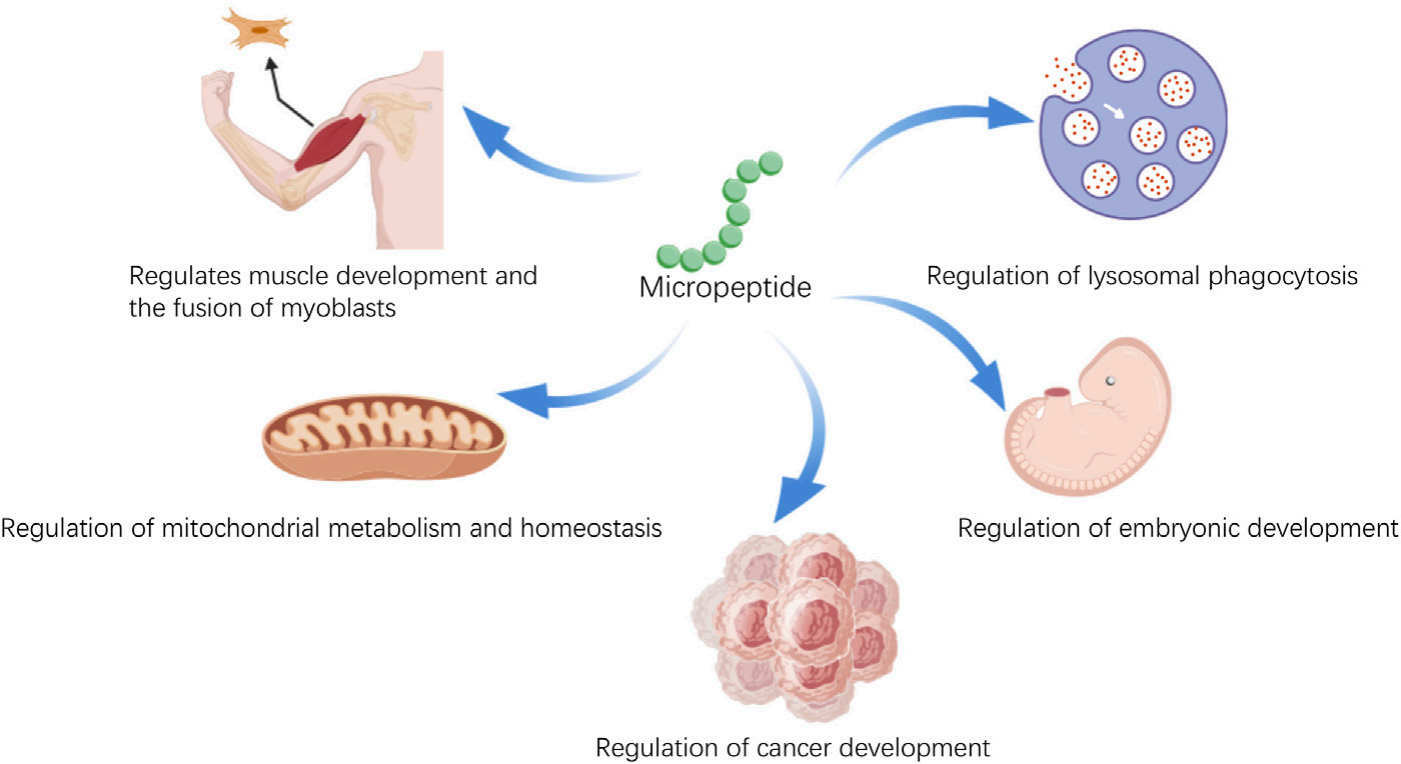
The various physiological functions of micropeptides.

Micropeptides, after undergoing post-translational modifications, can interact with other proteins, thereby exerting biological functions. As scientific technology advances, research into the biological functions of micropeptides is deepening. Studies have found that micropeptides play not only a crucial role in the life activities of organisms but also have significant implications in disease (Figure 1). Micropeptides have been shown to regulate muscle function by participating in calcium ion transport (Anderson et al., 2015) and the regeneration process of muscles (Nelson et al., 2016). They can help reduce inflammation by affecting the antigen presentation process of dendritic cells (Jackson et al., 2018); they are involved in the metabolic processes of mitochondria (Makarewich et al., 2018), impacting energy metabolism balance and fatty acid metabolism. In terms of the regulation of glucose and lipid metabolism: micropeptides are involved in the regulation of glucose and lipid metabolism, which helps to improve insulin resistance, reduce blood cholesterol levels, and promote the oxidation of fatty acids (Banerjee et al., 2020; Li et al., 2021). These functions may provide new therapeutic directions for treating cardiovascular diseases caused by lipid metabolism abnormalities and heart diseases caused by energy metabolism irregularities. In the field of tumor biology: micropeptides can regulate the proliferation rate and cell cycle progression of tumor cells. They inhibit tumor development by affecting glucose metabolism and ATP production (Huang et al., 2017; Pang et al., 2020; Wu et al., 2020). It can be seen that the biological functions of micropeptides are very rich, and research on micropeptides can help us better understand ourselves and provide better research directions for some intractable diseases. In this review, we describe the classification and distribution of micropeptides, their biological functions and mechanisms, and highlight their potential as diagnostic markers and therapeutic drugs.

## 2 Identification technologies for endogenous micropeptides

As research into the functions of micropeptides continues to advance, their crucial roles in various biological processes are gradually being unveiled. However, the identification and study of micropeptides still face numerous challenges. These small molecular peptides, encoded by long non-coding RNA (lncRNA), possess multiple biological functions and play a key role in the regulation of homeostasis, the onset and progression of diseases and cancers, and embryonic development. Detecting non-coding RNA through methods like qPCR is challenging due to non-specificity and the inability to reflect the levels of peptides that affect physiological functions. The following will introduce several methods for detecting micropeptides.

Western blot (WB) and Mass Spectrometry (MS) are standard techniques for protein detection, while Ribosome Profiling (Ribo-Seq) and Poly-Ribo-Seq are methods for identifying small open reading frames (sORFs) that translate micropeptides. The following is a summary of the advantages and disadvantages of these methods.

### 2.1 Western blot

Pros: A conventional and widely utilized technique, it offers reliable qualitative and semi-quantitative analysis of proteins and can detect the expression levels of specific proteins. Cons: It may not be effective for small molecular weight micropeptides due to limited antigenic sites and low expression, requiring the integration of gene editing techniques to enhance detection efficiency, which increases experimental complexity (Makarewich and Olson, 2017).

### 2.2 Mass spectrometry

Pros: It is high-throughput, capable of identifying numerous proteins and peptides, and is highly effective in identifying micropeptides and their interacting proteins, serving as a gold standard in proteomics (Fabre et al., 2021). Cons: Due to their low abundance and susceptibility to degradation, micropeptides require specialized enrichment and extraction processes that could lead to their loss or destruction. Additionally, there are high equipment costs, stringent technical demands, and complex data analysis.

### 2.3 Ribo-Seq

Pros: It can directly identify micropeptides in the translation process, offering direct evidence of protein synthesis. Cons: It is less effective in identifying smaller ORFs, potentially failing to accurately identify all micropeptides (Ingolia et al., 2009; Wilson and Masel, 2011).

### 2.4 Poly-Ribo-Seq

Pros: By analyzing fragments bound by multiple ribosomes, it can more precisely identify the sORFs of micropeptides in translation, providing more detailed information on translation efficiency compared to Ribo-Seq. Cons: The technique is complex and requires specialized bioinformatics analysis. It may also necessitate a substantial amount of starting material, which could be problematic for rare or difficult-to-culture samples (Aspden et al., 2014; Ji et al., 2015; Liang et al., 2018).

Each method has its unique applications and limitations. The choice of detection method should take into account the goals of the experiment, the nature of the sample, and the available resources and technologies. Often, a combination of methods can yield more comprehensive and accurate outcomes.

## 3 Types and functions of endogenous micropeptides in organisms

The micropeptides play a diverse range of roles in life activities; they are involved in various cellular processes such as growth, development, metabolism, and signal transduction. Endogenous micropeptides are widely present in a variety of human cells and tissues, including the nervous system, endocrine system, and immune system, influencing the course of human life activities. Based on the fundamental biological pathways of micropeptides, they can be roughly categorized into the following types (Table 1).

**TABLE 1:** Representative endogenous micropeptides of organisms.

Category	Micro-peptide	Mechanism of action
Micropeptides related to calcium ion homeostasis	ELN	Affecting Ca^2^ + Homeostasis
	MLN	Decreases the affinity of SERCA for Ca^2+^, reducing the contractility of muscle cells
	ALN	Affecting Ca^2^ + Homeostasis
	Scl	Affecting Ca^2^ + Homeostasis
	DWORF	Affecting Ca^2^ + Homeostasis
Mitochondrial Metabolism-Related Micropeptides	MOXI	Enhances the beta-oxidation of fatty acids, regulating mitochondrial metabolic efficiency
Myoblast Fusion and Muscle Development-Related Micropeptides	Myomixer	Promotes myoblast fusion, facilitating the merging of fibrocytes and myoblasts
Embryo Development-Related Micropeptides	Pri	Regulates the role of F-actin in epithelial morphogenesis, affecting the structure of the fruit fly’*s epidermis*
Material Degradation-Related Micropeptides	Hemotin	Modulates phagocytosis, participating in the degradation of intracellular materials
Cancer-Related Micropeptides	CASIMO1	Interacts with SQLE, regulating the metabolic homeostasis of cancer cells and affecting proliferation

### 3.1 Micropeptides related to calcium ion homeostasis

Micropeptides associated with the sarcoplasmic/endoplasmic reticulum (SERCA) have a significant impact on the intracellular calcium ion ([Ca] ^ (2+)) homeostasis. SERCA is a membrane pump that transports [Ca]^2+^ from the cytoplasm to the sarcoplasmic reticulum, regulating the calcium ion concentration within muscle cells, thereby controlling the contraction and relaxation states of muscles (Anderson et al., 2015), and influencing the physiological functions of muscle physiology. Studies have found that micropeptides such as ELN (Anderson et al., 2016), MLN (Anderson et al., 2015), ALN (Anderson et al., 2015), Scl (Magny et al., 2013), and DWORF (Nelson et al., 2016) play an important role in this process. Among them, MLN, a 46-amino acid micro-peptide encoded by a skeletal muscle-specific long non-coding RNA. MLN is primarily localized in the sarcoplasmic reticulum membrane of muscle cells, reducing the affinity of SERCA for [Ca]^2+^ by interacting with SERCA, thereby reducing the intake of [Ca]^2+^ and decreasing the contractility of muscle cells. These micropeptides not only affect muscle cells but also regulate [Ca]^2+^ homeostasis in non-muscle cells, playing an important role in functions such as cell contraction, growth, and metabolism (Anderson et al., 2015).

### 3.2 Mitochondrial metabolism-related micropeptides

These micropeptides are involved in mitochondrial metabolic activities. For instance, the MOXI micro-peptide (Makarewich et al., 2018) acts as a key regulator of mitochondrial metabolism. MOXI is a micropeptide encoded by nuclear genes and is primarily located in the mitochondrial inner membrane (Makarewich et al., 2018). It enhances the beta-oxidation process of fatty acids by binding to the mitochondrial trifunctional protein to form an essential enzyme complex. In animal experiments, the knockout of MOXI led to weakened fatty acid metabolism in cardiac and muscular mitochondria, which in turn reduced exercise capacity. This indicates that MOXI plays a significant role in regulating the efficiency of mitochondrial metabolism (Makarewich et al., 2018).

### 3.3 Myoblast fusion and muscle development-related micropeptides

These micropeptides are involved in the growth and development of muscle tissue. For example, the Myomixer micro-peptide (Bi et al., 2017) is a micropeptide encoded by nuclear genes and is primarily distributed in the cell membrane and mitochondria (Wang et al., 2020). Specifically, its localization in the cell membrane may be related to its function in promoting the fusion of muscle cells. The specific micro-peptide myomixer plays a key role in the differentiation of myoblasts. Myomixer promotes the fusion of myoblasts and binds to the fusion membrane protein myomaker, together facilitating the fusion between fibroblasts and myoblasts (Bi et al., 2017), thereby influencing muscle development.

### 3.4 Embryo development-related micropeptides

These micropeptides are involved in the normal development of embryos. For example, the Pri micro-peptide. Kondo et al. discovered in the fruit fly’s epithelial tissue a micro-peptide Pri encoded by lncRNA polished rice (pri), which consists of either 11 or 32 amino acids and plays an important role in regulating F-actin during epithelial morphogenesis. The loss of Pri function leads to the disappearance of the fruit fly’s epidermal structure (Kondo et al., 2007).

### 3.5 Material degradation-related micropeptides

These micropeptides participate in the degradation of intracellular materials, capable of breaking down toxins and waste produced by cellular metabolism, such as Hemotin (Pueyo et al., 2016). Hemotin is primarily localized in the early endosomes of *Drosophila* hemocytes (Pueyo et al., 2016). As a transmembrane micropeptide, Hemotin regulates phagocytosis by modulating the maturation process of endosomes. Hemotin is a tissue-specific sORF gene, which encodes an 88-amino acid transmembrane micro-peptide called Hemotin. Hemotin is mainly expressed in fruit fly macrophages and can regulate phagocytosis (Pueyo et al., 2016).

### 3.6 Cancer-related micropeptides

These micropeptides are involved in the occurrence and development of cancer. For example, CASIMO1 and MIAC. CASIMO1 (cancer-associated small integral membrane open reading frame 1): It is the first functional micro-peptide found to have an oncogenic effect. CASIMO1 is primarily localized in the endosomes within cells (Zhou et al., 2024). It interacts with the key enzyme of cholesterol synthesis, squalene epoxidase (SQLE), to regulate the metabolic homeostasis of cancer cells, thereby influencing cell proliferation and cell cycle progression, and regulating lipid droplet accumulation in breast cancer cells. Knocking down CASIMO1 can lead to a reduction in the proliferation of breast cancer cells (Polycarpou-Schwarz et al., 2018).

## 4 Endogenous micropeptides as disease diagnostic biomarkers and potential drugs

### 4.1 Micropeptides as biomarkers for disease diagnosis

The assessment of diseases can be facilitated through the detection of micropeptides. Melanoma is an aggressively malignant form of skin cancer. Between 2008 and 2010, scientists discovered two new micropeptides, MELOE-1 and MELOE-2 (Charpentier et al., 2022), within melanoma cells. These micropeptides are produced through IRES-dependent translation of the long non-coding RNA meloe. The discovery of these micropeptides has provided a new perspective for the treatment of melanoma. Further research has revealed the potential link between MELOE-1 and MELOE-2 and the prevention of melanoma patient relapse. A study by Godet (Godet et al., 2008) and colleagues showed that among melanoma patients treated with tumor-infiltrating lymphocytes (TILs) containing MELOE-1 specific T-cells, the proportion of patients without relapse was significantly higher than that of patients who did relapse. Additionally, T-cells reactive to MELOE-2 were also found in patients who did not relapse after TIL treatment.

These findings suggest that melanoma antigens encoded by meloe may participate in the T-cell immune surveillance process (Godet et al., 2010), helping to reduce the risk of patient relapse. This mechanism offers a new strategy for the immunotherapy of melanoma, which involves enhancing or mimicking the immune response of T-cells to tumor antigens to prevent disease recurrence. Future research will need to further verify the specific role of these micropeptides in the immune response to melanoma and explore their potential as therapeutic targets.

Colorectal Cancer (CRC) is a common malignant tumor in the gastrointestinal tract. Recent research has found that micropeptides encoded by long non-coding RNA (lncRNA) play a significant role in the occurrence and development of CRC, acting as either tumor suppressors or oncogenes. In 2017, Huang et al. discovered a micropeptide encoded by lncRNA HOXB-AS3 (Huang et al., 2017) in CRC tissue. This micropeptide competitively binds to heterogeneous nuclear ribonucleoprotein A1 (hnRNP A1), inhibiting PKM splicing regulation mediated by hnRNP A1, and thus regulating the process of cancer metabolic reprogramming, affecting the malignant progression of tumors. Also in the same year, another micropeptide, FORCP, encoded by LINC00675, was found. It modulates CRC cell apoptosis in response to endoplasmic reticulum stress, exerting a tumor-suppressing effect (Li et al., 2020). The expression levels of these micropeptides may be associated with the malignant progression of colorectal cancer (CRC), thus they can serve as potential diagnostic and prognostic markers. In the treatment of colorectal cancer, the micropeptide ASAP encoded by LINC00467 has significant research value as a potential diagnostic and prognostic marker. ASAP promotes the proliferation of colorectal cancer cells by directly modulating the activity of ATP synthase, thereby affecting the malignant progression of tumors (Ge et al., 2021). ASAP interacts with the α and γ subunits of ATP synthase (ATP5A and ATP5C), enhancing the construction of ATP synthase, increasing the activity of ATP synthase and the oxygen consumption rate of mitochondria, and thus promoting the proliferation of CRC cells. Furthermore, high expression levels of ASAP and LINC00467 are closely related to the poor prognosis of CRC patients. In clinical samples, the expression level of ASAP in CRC tissue is higher than that in matched adjacent non-cancerous tissue, and tumors with higher ASAP expression show relatively high levels of Ki-67, ATP5A, and ATP5C. This suggests that ASAP may serve as a candidate drug molecule for CRC, and its expression level may be used to predict patient prognosis. The study also found that ASAP is upregulated in CRC tissue and is associated with poor patient prognosis. This further confirms the potential of ASAP as a diagnostic and prognostic marker. Therefore, the discovery of ASAP provides a new perspective for the diagnosis and treatment of colorectal cancer and may become an important target for CRC treatment in the future.

Breast cancer research continues to delve deeper, revealing the significant role of lncRNA-encoded micropeptides in regulating the biological behavior of tumors. The discovery of the CASIMO1 micropeptide by Polycarpou-Schwarz (Polycarpou-Schwarz et al., 2018) and colleagues in 2018 marked an important advancement in the study of breast cancer micropeptides, providing a new perspective for understanding the lipid metabolism regulation in hormone receptor-positive breast cancer. This micropeptide is not only functional within tumor cells but may also affect metabolic interactions in the tumor microenvironment, offering potential for the development of new metabolic targeting therapeutic strategies. In 2022, the research on the PACMP micropeptide by Zhang (Zhang et al., 2022) and colleagues further highlighted the role of DNA damage response in the resistance to breast cancer treatment. The discovery of the PACMP micropeptide suggests that modulating DNA repair mechanisms may offer new avenues for overcoming tumor drug resistance (Zhang et al., 2022). This could involve inhibitors targeting specific DNA repair proteins or the use of combined chemotherapy and radiotherapy to enhance therapeutic effects.

Research into triple-negative breast cancer (TNBC), a more aggressive subtype of breast cancer, has identified micropeptides such as ASRPS and XBP1SBM that play a key role in tumor angiogenesis and metastasis (Wu et al., 2022). The identification of these micropeptides has not only improved our understanding of TNBC’s aggressiveness but also provided potential targets for developing new angiogenesis inhibitors. Moreover, therapeutic strategies targeting these micropeptides may need to incorporate anti-angiogenic therapies and immunotherapies to achieve a more comprehensive treatment effect on TNBC. Research on the TGF-β signaling pathway has revealed its complex role in the metastasis of breast cancer (Guo et al., 2020). The interaction between the CIP2A-BP micropeptide, encoded by LINC00665, and the TGF-β signaling pathway suggests that targeting the TGF-β signaling pathway or its downstream effectors may effectively suppress the translational inhibition of CIP2A-BP, thereby inhibiting the malignant progression of tumors. It is hoped that further translational medical research will improve the treatment outcomes for breast cancer patients.

Hepatocellular carcinoma (HCC) is a primary liver malignancy. In 2020, Pang et al. discovered that numerous lncRNAs in cancer cells have the potential to bind with ribosomal protein S6 (RPS6) and encode micropeptides (Pang et al., 2020). Among these, LINC00998 has garnered significant attention due to its ability to encode a conserved 59-amino acid peptide, SMIM30 (Pang et al., 2020), which promotes HCC tumorigenesis by regulating cell proliferation and migration independently of the lncRNA itself. Another independently functioning micropeptide, KRASIM, encoded by lncRNA NCBP2-AS2, has been identified as a novel microprotein inhibitor of the KRAS pathway. KRASIM interacts with the Kirsten rat sarcoma viral oncogene homolog (KRAS) to reduce KRAS protein levels, thereby inhibiting the transmission of oncogenic signals in HCC cells.

Inducing apoptosis in cancer cells is another significant way micropeptides can suppress the progression of malignancy. A micropeptide encoded by lncRNA HBVPTPAP modulates the JAK/STAT signaling pathway to induce HCC cell apoptosis, thus inhibiting the malignant progression of HCC (Lun et al., 2020). While many lncRNAs involved in human cancer progression have been shown to encode biologically active peptides, the role of lncRNA-encoded micropeptides in HCC cell senescence remains largely unknown. Xiang et al. (2021) reported a micropeptide, PINT87aa, encoded by LINC-PINT, which plays an important role in HCC cell senescence. PINT87aa is significantly upregulated in a hydrogen peroxide-induced HCC cell senescence model, and its overexpression can induce growth arrest, cellular senescence, and reduce mitochondrial autophagy. Furthermore, the second exon of LINC-PINT can form a circular molecule, circPINT, which encodes the functional micropeptide PINT87aa. This micropeptide can directly interact with the polymerase-associated factor complex (PAF1c) to inhibit the transcriptional elongation of multiple oncogenes, thereby suppressing the proliferation of glioblastoma tumor cells. These findings indicate that LINC-PINT plays a crucial role in the occurrence and development of cancer and could serve as a biomarker for future cancer therapy and prognosis. The exploration of the specific mechanisms of action of these micropeptides and their potential in HCC treatment is a promising direction for future research.

Esophageal squamous cell carcinoma (ESCC) is the most common pathological type of esophageal cancer, accounting for approximately 90% of cases and represents a clinically serious disease. However, the association between ESCC and micropeptides encoded by long non-coding RNAs (lncRNAs) remains largely unknown. In 2020, Wu et al.characterized a Y-linked lncRNA, LINC00278, in male ESCC (Wu et al., 2020). The first exon of this lncRNA contains a small open reading frame (sORF) that encodes a micropeptide, Yin Yang 1 binding micropeptide (YY1BM), which is a potential anti-tumor factor. The smoking-induced protein ALKBH5 can demethylate the m6A of lncRNA, thereby reducing the translation efficiency of the sORF of LINC00278 (Wu et al., 2020). Furthermore, YY1BM is involved in the progression of ESCC by inhibiting the interaction between YY1 and the androgen receptor (AR), leading to a decrease in the expression of eukaryotic elongation factor-2 kinase (eEF2K) through the AR signaling pathway. Consequently, the downregulation of YY1BM can significantly increase eEF2K expression and inhibit apoptosis, enabling ESCC cells to adapt better to nutrient-deficient conditions (Banday et al., 2020).

These results suggest a mechanistic link between smoking and AR signaling in the progression of male ESCC. The risk of cancer in males increases when the smoking-induced demethylation of Y chromosome-associated lncRNA m6A occurs. This insight underscores the potential role of smoking-induced epigenetic changes in the development of ESCC, particularly in male patients, and highlights the importance of further research into the regulatory mechanisms involving lncRNA-encoded micropeptides in cancer progression.

**FIGURE 2:**
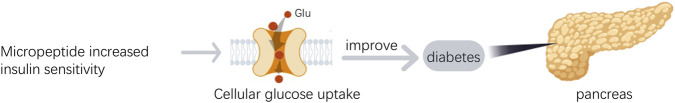
The role of micropeptides in diabetes.

MOTS-c, a micropeptide derived from mitochondrial 12S rRNA, composed of 16 amino acids, is capable of migrating to the nucleus and regulating gene expression in response to metabolic stress. Research indicates that MOTS-c is involved in the regulation of intracellular glucose and fatty acid metabolism, and is associated with insulin resistance and obesity (Cataldo et al., 2018). By affecting purine nucleotide synthesis and the folate cycle, MOTS-c contributes to the improvement of insulin sensitivity (Figure 2) and the regulation of fat metabolism (Lee et al., 2015). During this process, MOTS-c leads to a decrease in 5-methyltetrahydrofolate levels, while increasing the levels of folate, methionine, and homocysteine. Furthermore, MOTS-c promotes an increase in the levels of 5-aminoimidazole-4-carboxamide ribonucleotide (AICAR), which activates the AMP-activated protein kinase (AMPK) signaling pathway, promoting fatty acid oxidation and glucose uptake, thereby enhancing mitochondrial function and maintaining glucose homeostasis (Lee et al., 2015). The expression levels of MOTS-c are negatively correlated with body mass index (BMI), fasting insulin, and glycated hemoglobin levels, suggesting its potential role in diabetes management (Cobb et al., 2016). Another micropeptide, SHLP2, can activate extracellular signal-regulated kinase (ERK) and signal transducer and activator of transcription-1 (STAT-1), promoting cell proliferation and energy expenditure, and increasing ATP production, thus enhancing mitochondrial function and glucose metabolism. In animal experiments, the injection of SHLP2 has increased glucose uptake and inhibited hepatic glucose production (Hashimoto et al., 2001). HN peptide, another mitochondria-derived micropeptide, promotes insulin release and improves glucose sensitivity by increasing the translocation of glucose transporter 2 (GLUT2) and the activity of glucokinase (GCK). HN analogs can also promote insulin secretion and glucose uptake by enhancing the phosphatidylinositol 3-kinase (PI3K)/protein kinase B (AKT) signaling pathway and signaling molecules in fatty acid metabolism, reducing hepatic glucose production, offering new strategies for the treatment of diabetes (Wu et al., 2021). These findings reveal the important role of micropeptides in diabetes and metabolic diseases, providing new directions for future treatment. As research progresses, these micropeptides may become candidate drug molecule for the treatment of diabetes.

The pathogenesis of Alzheimer’s disease (AD) is closely related to mitochondrial dysfunction (Yao et al., 2009), and the brain is one of the tissues with the highest content of mitochondria. HN peptide, initially discovered in the occipital lobe of AD patients, has been found to have reduced expression in the hippocampus of rats under the influence of ovarian hormone deprivation, potentially serving as a biomarker for mitochondrial dysfunction in AD (Zárate et al., 2019). As age increases, the levels of HN in the serum of humans and mice decrease, yet HN can enhance insulin sensitivity. In the central nervous system, newly developed potent HN derivatives can improve central insulin sensitivity when administered intravenously (Muzumdar et al., 2009). The progression of AD is associated with changes in glucose metabolism in the brain. Impairment of the insulin-mediated AKT signaling pathway leads to increased enzyme activity of GSK-3β, which in turn causes phosphorylation of tau protein, a key pathological feature of AD (Zhang et al., 2018). Micropeptides are capable of modulating insulin secretion, sensitivity, and mitochondrial function, and these regulatory sites may become potential therapeutic targets for improving cognitive function in AD patients (Potenza et al., 2021). SHLP2 is a micropeptide that enhances peripheral insulin sensitivity and prevents neuron cell death due to LDH leakage in AD models (Cobb et al., 2016). Additionally, SHMOOSE, encoded by a small ORF on the mitochondrial serine tRNA, is involved in brain energy metabolism by regulating mitochondrial gene expression. The levels of SHMOOSE in cerebrospinal fluid are correlated with the pathological features of AD, and intracerebroventricular administration of SHMOOSE can regulate mitochondrial gene expression in the brain, increase mitochondrial reserve capacity during cellular stress, optimize proton flow through the mitochondrial inner membrane, and increase mitochondrial oxygen consumption (Miller et al., 2023).

### 4.2 The application of endogenous micropeptides as potential drugs

Micropeptides are short polypeptide chains formed by the linkage of amino acids through peptide bonds. They possess high biocompatibility, extended half-life within the body, strong tissue penetration, and low toxicity, which facilitates their synthesis and modification, and they hold significant potential as candidate drug molecule (Table 2). The application potential of the micropeptide MOTS-c in the field of diabetes treatment is increasingly being recognized by the medical research community. The latest research indicates that MOTS-c effectively regulates key metabolic processes such as glucose metabolism, lipid metabolism, and bone metabolism by promoting fatty acid oxidation, browning of white fat, enhancing glucose utilization, and improving insulin sensitivity, which is particularly crucial for the management of type 2 diabetes (Wu et al., 2019). Additionally, in models of type 1 diabetes, MOTS-c supports the function of regulatory T cells, protecting islet cells from autoimmune attacks and maintaining normal insulin production (Kong et al., 2021). In the treatment of diabetic cardiomyopathy, MOTS-c improves cardiac function and structural abnormalities by activating the AMPK signaling pathway and inhibiting inflammatory responses, while also exerting antioxidant effects, increasing the activity of antioxidant enzymes, and reducing oxidative stress in cardiomyocytes (Wu et al., 2023). MOTS-c can also repair myocardial damage by regulating the CCN1/ERK1/2/EGR1 signaling pathway, providing a new therapeutic target for the management of diabetes complications. Although MOTS-c has shown positive effects in animal models, its potential for application in human diabetes treatment still requires further validation through clinical studies (Wang et al., 2022). Future research will explore the efficacy and safety of MOTS-c in diabetes treatment and investigate how to translate it into actual clinical applications. These biological functions of MOTS-c offer broad prospects for its application in diabetes treatment and it is expected to become a candidate drug molecule for the treatment of diabetes and its complications.

**TABLE 2:** Representative endogenous micropeptides as potential drugs.

Micropeptide name	Target disease	Peptide length	Mechanism of action
MOTS-c	Diabetes, Obesity	Composed of 16 amino acids	Regulates purine nucleotide synthesis and the folate cycle, improves insulin sensitivity, regulates fat metabolism
HN	Alzheimer’s disease	Composed of 24 amino acids	Enhances insulin sensitivity, provides neuroprotection, inhibits apoptosis
MIAC	Head and neck squamous cell carcinoma	Composed of 51 amino acids	Interacts with aquaporin-2 (AQP2), inhibits tumor growth and progression
PINT87aa	Liver cancer	Composed of 87 amino acids	Induces cellular senescence, inhibits tumor cell proliferation

**FIGURE 3:**
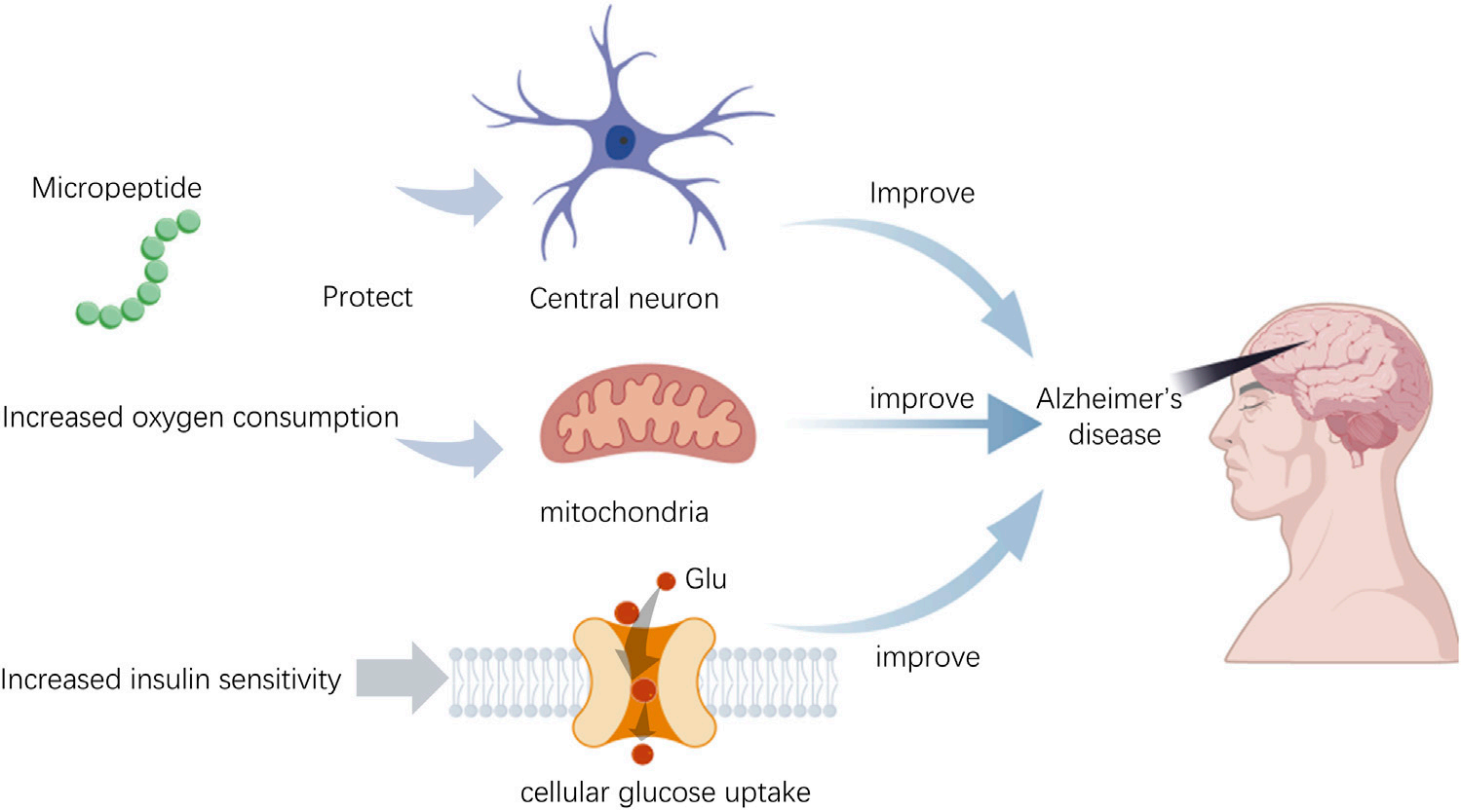
The role of micropeptides in Alzheimer’s disease.

Micropeptide HN (Humanin) demonstrates multifaceted potential applications in the treatment of Alzheimer’s disease (AD), with mechanisms of action that include neuroprotection, antioxidant and anti-inflammatory effects, regulation of insulin sensitivity, inhibition of apoptosis, and maintenance of mitochondrial function (Figure 3). Specifically, HN can counteract neuronal damage caused by AD-related factors, particularly the toxicity of beta-amyloid proteins (Aβ). It has been shown *in vitro* to protect neurons from Aβ toxicity and has demonstrated improved effects on memory deficits in animal models (Yen et al., 2018). Furthermore, HN reduces oxidative stress by enhancing the activity of antioxidant enzymes, thereby protecting neuronal cells (Niikura, 2022). HN also improves insulin sensitivity, which is crucial for enhancing cognitive function in AD patients, as insulin resistance is closely related to the development of AD (Niikura, 2022). By modulating the JAK/STAT signaling pathway, HN inhibits apoptosis and reduces neuronal cell death. Lastly, HN helps maintain the structure and function of mitochondria, which is essential for the survival and proper functioning of neuronal cells (Niikura, 2022). These characteristics position HN as a promising candidate drug molecule for the development of new therapies aimed at AD and its associated complications.

In cancer treatment, micropeptides have shown immense potential. They offer high specificity and activity, low cytotoxicity, and low immunogenicity, positioning them as attractive candidates for targeted therapy. Preclinical research has already begun for some micropeptides in cancer treatment. For instance, research has identified a micropeptide encoded by the lncRNA HOXB-AS3 that inhibits the growth of colon cancer. A micropeptide named MIAC (Pan et al., 2022), discovered by Xu (Li et al., 2022) and colleagues, binds to AQP2 and suppresses the EREG/EGFR signaling pathway, thereby inhibiting the progression and metastasis of renal cell carcinoma.

The micropeptide PINT87aa, encoded by the long non-coding RNA LINC-PINT, has shown potential applications in the treatment of hepatocellular carcinoma (HCC). According to research by Xiaohong Xiang and colleagues published in the journal Theranostics, PINT87aa induces cellular senescence in liver cancer cells by blocking FOXM1-mediated PHB2, thereby inhibiting the proliferation of tumor cells and promoting cellular senescence. This discovery provides a candidate drug molecule for the treatment of HCC (Xiang et al., 2021). PINT87aa is significantly upregulated in a hydrogen peroxide-induced HCC cellular senescence model. Overexpression of PINT87aa can inhibit cell growth, induce cellular senescence, and reduce mitophagy. Furthermore, PINT87aa is able to directly bind to FOXM1, affecting its transcriptional activity, and subsequently downregulating the expression of PHB2, a protein involved in mitophagy. These research findings suggest that PINT87aa is not only a novel biomarker for cellular senescence but also a key regulatory factor, offering new strategies for the treatment of HCC.

In summary, these examples demonstrate that micropeptides have significant potential in disease detection and treatment and are a hot topic in biomedical research. With further research and technological advancements, micropeptides are expected to become important tools for the early diagnosis and treatment of diseases.

## 5 Future research directions and challenges

The swift advancement of large-scale genomic sequencing has expedited our understanding of the genome, unveiling the intricate nature of sORF sequences. The revelation of micropeptides underscores their critical importance in biological functions and their pivotal regulatory roles in both life processes and the progression of diseases. There is a growing interest among researchers in ncRNA-encoded peptides that partake in the genesis and evolution of cancer, potentially offering novel insights into the mechanisms underlying malignant diseases. Furthermore, the low cytotoxicity and immunogenicity of ncRNA-encoded peptides make them promising candidates for exploring new cancer therapeutics. Despite the existence of various biotechnologies capable of unearthing unknown micropeptides, their small size and low expression levels present technical challenges, leaving many micropeptides yet to be discovered. It is anticipated that with the ongoing expansion and refinement of technologies and methodologies, these hurdles will be surmounted in forthcoming research endeavors. Concurrently, significant efforts are necessary to delve into the biological functions and mechanisms of micropeptides to foster their application in physiological processes and the clinical management of diseases.
